# Preliminary Evidence of Reduced Urge to Cough and Cough Response in Four Individuals following Remote Traumatic Brain Injury with Tracheostomy

**DOI:** 10.1155/2016/6875210

**Published:** 2016-09-27

**Authors:** Erin Silverman, Christine M. Sapienza, Sarah Miller, Giselle Carnaby, Charles Levy, Hsiu-Wen Tsai, Paul W. Davenport

**Affiliations:** ^1^Division of Pulmonary, Critical Care, and Sleep Medicine, Department of Medicine, College of Medicine, University of Florida, Gainesville, FL, USA; ^2^Brooks Rehabilitation College of Health Sciences, Jacksonville University, Jacksonville, FL, USA; ^3^Brooks Rehabilitation Hospital, Brooks Health, Jacksonville, FL, USA; ^4^College of Nursing, Medical University of South Carolina, Charleston, SC, USA; ^5^Department of Communication Sciences and Disorders, University of Central Florida, Orlando, FL, USA; ^6^Physical Medicine and Rehabilitation Service, North Florida/South Georgia Veterans Health System, Gainesville, FL, USA; ^7^Veteran's Health Administration, Center for Innovation in Disability and Rehabilitation Research, Gainesville, FL, USA; ^8^Department of Physiological Sciences, College of Veterinary Medicine, University of Florida, Gainesville, FL, USA

## Abstract

Cough and swallow protect the lungs and are frequently impaired following traumatic brain injury (TBI). This project examined cough response to inhaled capsaicin solution challenge in a cohort of four young adults with a history of TBI within the preceding five years. All participants had a history of tracheostomy with subsequent decannulation and dysphagia after their injuries (resolved for all but one participant). Urge to cough (UTC) and cough response were measured and compared to an existing database of normative cough response data obtained from 32 healthy controls (HCs). Participants displayed decreased UTC and cough responses compared to HCs. It is unknown if these preliminary results manifest as a consequence of disrupted sensory (afferent) projections, an inability to perceive or discriminate cough stimuli, disrupted motor (efferent) response, peripheral weakness, or any combination of these factors. Future work should attempt to clarify if the observed phenomena are borne out in a larger sample of individuals with TBI, determine the relative contributions of central versus peripheral nervous system structures to cough sensory perceptual changes following TBI (should they exist), and formulate recommendations for systematic screening and assessment of cough sensory perception in order to facilitate rehabilitative efforts. This project is identified with the National Clinical Trials NCT02240329.

## 1. Introduction

A spectrum of airway defense mechanisms, including reflex laryngoadduction, cough, and swallow, protect the lungs from aspiration of foreign material. Stimulus-induced cough (sometimes referred to as “reflexive cough”) is the focus of this investigation. Stimulus-induced cough can be both mechanosensory and chemosensory in nature. Cough in response to mechanosensory inputs is not chemically reactive or responsive to capsaicin. This type of cough may prevent aspiration of gastric material or particulates into the lungs [1]. Chemosensory cough is mediated, in part, through the action of unmyelinated C fibers, preventing respiratory disease or irritant-induced lung injury [1, 2]. Cough removes mucus, fluid, and airway obstructions or irritants. Cough is both a subcortical and cortical neural process, and perception of airway stimuli is cortically mediated in all but extreme coma states [2–5]. Interoceptive respiratory sensation is a state-dependent phenomenon, with cerebral cortical activity quantified by a respiratory-related evoked potentials (RREP) elicited following airway stimulation [4].

Cortical perfusion is altered secondary to edema and elevation of intracranial pressures following traumatic brain injury (TBI), potentially contributing to the emergence or worsening of depressive symptoms [5] as well as interfering with sensorimotor modalities anywhere along a continuum from mild to severe, typically corresponding to the severity of the injury [6].

Airway defensive mechanisms, including cough and swallow, are also frequently impaired following TBI [7–11]. Other comorbidities associated with TBI include laryngeal disorders and cricopharyngeal dysfunction [10, 11]. Of these, swallow dysfunction or* dysphagia*, as well as its potential contribution to the development of aspiration-related lung infection following TBI, is well documented [8–11]. However, aside from clinical observations of attenuated cough responses to penetration or aspiration of food or liquid administered during clinical swallow assessments, no information exists as to the manner in which stimulus-induced cough is potentially altered following TBI. Also unknown are the relative contributions of disordered peripheral or central sensory structures to these processes, or the interplay among sensory perception, cognitive/affective awareness of cough stimuli, and motor response following TBI, particularly given the fact that neuromotor systems are almost universally affected. Current evidence suggests that both voluntary and reflexive peak cough airflows are significantly reduced in those with TBI, relative to normative values [7].

Typically, mechanical and chemical irritation of the airways alerts cortical sensory mechanisms responsible for sensory discrimination, eliciting an “urge to cough” (UTC). UTC is immediately followed by a motor cough response that protects the airway by expelling aspirated foreign material [12]. It is reasonable to assume that if the capacity for sensory perception and discrimination is impaired, then the risk of uncompensated aspiration and resultant lung infection increases [12].

Aspiration is common and frequently silent (aspiration followed by no cough response) in almost half of all individuals with TBI [8]. The consequences of aspiration are potentially life-threatening [13, 14]. Compared to the general population, individuals with TBI are over two times more likely to die from serious medical complications. Chief among these is aspiration pneumonia, even when controlling for age, gender, and race [15]. At one year after TBI, individuals are 49 times more likely to die of aspiration pneumonia and four times more likely to die of (nonaspiration) pneumonia than the general population [16]. These findings support the theory that airway defense mechanisms are impaired following TBI. High observed rates of aspiration-related lung infections in those with TBI [8] may be a consequence of disordered cough (*dystussia*) in this vulnerable patient group. Cough, as a clinical indicator of airway compromise during eating, was observed during one investigation to be present in 44% of all patients with TBI [7]. It is unknown to what extent both the perception of UTC and the cough response itself are altered by TBI, including to what extent dampening of sensory inputs, central processing of these inputs, disrupted motor pathways, and/or peripheral weakness/atrophy contribute to observed* dystussia* symptoms.

Stimulus-induced cough is experimentally elicited by inhaling an aerosolized tussive stimulus such as capsaicin. One measure of sensitivity to a tussive stimulus is the concentration required to elicit a minimum of two coughs (C2) [3, 17–21]. Individuals with reduced stimulus-induced cough responses require greater concentrations of the tussive agent to elicit C2 [3, 17–21]. Once a stimulus-induced cough response occurs, the* cognitive sensation* of UTC can also be determined [22]. UTC is a sensory perceptual response which manifests immediately prior to cough execution and typically occurs at a lower concentration of a stimulus solution than that needed to produce a cough response. This means that, during low-threshold stimulation, the perceived* need* to cough may (or may not) preceed an actual cough response. An absent UTC may place an individual at increased risk of uncompensated aspiration. The UTC has both discriminative and affective (emotional) components [22] and is one of the respiratory-related interoceptive sensations critical for behavioral modification of the cough response itself [23]. For example, in social situations where coughing would be disrupted, the desire to cough can be actively suppressed or exchanged for a less disruptive throat clearing.

Finally, cough and swallow are interrelated on both a functional and a neuroanatomical level [24]. The coordinated actions of shared cough-swallow behavioral control assemblies exert influence over central pattern generators for breathing and airway defense [25]. Concordant deficits of cough and swallow have been observed within patient groups [26–28]. The objective of this study was to document cough perception and response in a small cohort of individuals following TBI. We hypothesized that individuals with TBI would demonstrate decreased UTC in response to stimulation, increased C2 threshold, and reduced total number of coughs (CTot) produced in response to inhalation of various concentrations of aerosolized capsaicin.

## 2. Methods

This project was approved by the University of Florida Health Science Center IRB and there was full adherence to all regulatory standards. All participants gave informed consent for participation.

Participants were recruited for this study through communication with a local hospital-based TBI support group. Nonsmoking men and women, 18 years of age or older, who had sustained a TBI within the preceding five years were considered for inclusion. Exclusion criteria included a history of extensive oral reconstruction or surgery, current (or within the preceding year) tracheostomy, or a history of alcohol or illicit substance abuse. Additional criteria for exclusion were chronic cough and/or cough syncope, head or neck cancer, history of radiation to the head or neck, moderate-to-severe chronic obstructive pulmonary disease, stroke, or other neurodegenerative diseases. All participants reported general neurological and medical stability at the time of enrollment. Following informed consent, general medical history (including events surrounding TBI and ensuing rehabilitation) was obtained via interview. Participants (and their family members where present) were asked about experiencing swallowing problems following TBI or not, the extent or severity of those problems, completing swallowing therapy or not, and current swallow status including diet consistencies. TBI severity was estimated through assignment of an appropriate Rancho Los Amigos Scale (RLAS) [29] score and administration of the Montreal Cognitive Assessment (MoCA) [30] by the lead clinician (experienced in the administration and interpretation of both measures). The RLAS is a global estimate of awareness, cognition, behavior, and interaction with the environment. The RLAS assigns a score along an ordinal scale ranging from nonresponsive (Level I) to purposeful and appropriate (Level VIII). The MoCA is a rapid screening of mild cognitive dysfunction that assesses attention and concentration, executive function, memory, language, visuoconstructional skills, conceptual thinking, calculation, and orientation. Possible scores range from zero to 30 and a score of 26 or greater is considered “normal.”

HC data was previously obtained, using identical procedures, by our laboratory. Inclusion and exclusion criteria for HC participants were identical to those of the participants with TBI (with the obvious exception of TBI diagnosis). Cough testing was completed with the participants in a seated position and mouth and nose covered by a standard disposable medical anesthesia facemask. This facemask was coupled to a pneumotachograph, pressure transducer, and nebulizer. The aerosols were delivered through a DeVilbiss T piece connected to a dosimeter programmed to deliver an aerosolized solution (delivery duration of two seconds) upon inhalation. Participants completed a capsaicin challenge with three randomized blocks of 0 (nonstimulus control solution), 50, 100, 200, and 500 *μ*M capsaicin dissolved in a base solution of 80% physiological saline and 20% ethanol [31]. Note that only participants with TBI (no HCs) were presented with an additional three presentations of concentration of 500 *μ*M capsaicin in solution (randomly presented among all other concentrations) in order to explore response to stimulation above 200 *μ*M, should responses be dampened in those with TBI. Cough airflow signals were relayed to a Dell computer with a Power lab Data Acquisition System (AD Instruments, Colorado Springs, CO).

Following 30 seconds of tidal breathing (to allow for acclimation to the facemask), participants were instructed to “take a quick (≤1 sec.) and sharp breath in” whereupon the nebulized capsaicin solution was automatically administered by the dosimeter. Following each aerosol presentation, the participant was instructed to rate their UTC using a modified Borg Rating Scale [32] where 1 = no UTC and 10 = maximum UTC. For Participant 1, who demonstrated severe visual impairment, the Borg Rating Scale was read aloud and the participant given time to respond. Participants were given sufficient time to practice using the Borg Scale to the point where the investigator administering the capsaicin solution felt confident that the instructions for UTC ratings were well understood and could be accurately executed. Between presentations, participants were given a minimum of a one-minute rest period where water was offered. Whether participants opted to drink water or not, the next aerosol presentation was delayed until the participant reported perceiving no residual irritation.

The total cough count (CTot) was determined by counting all cough events that occurred following each presentation of capsaicin solution. CTot was made, in real time, by two investigators and confirmed via review of the recorded cough airflow signal. Capsaicin concentration necessary to elicit a two-cough threshold response (C2) within 30 seconds of presentation, on at least two (out of three) trials of that concentration, was identified from the cough count record. For the purposes of this study, C2 was selected over alternative measures (such as C5) as a means of examining the lowest capsaicin concentration for cough elicitation.

Effect size measures for two independent groups (Cohen's *d*) were calculated for UTC and CTot at 50, 100, and 200 *μ*M capsaicin for participants with TBI and healthy control (HC) participants.

## 3. Results

Four participants with TBI (2 men and 2 women) completed this study and the results obtained were compared with an existing database of 32 HC (19 men and 13 women) participants. All were reportedly nonsmokers. Please refer to Table 1 for demographic data on each of the participants with TBI. At the time of enrollment, participants ranged from RLAS VI (confused-appropriate) to VIII (purposeful-appropriate). Median RLAS was Level VII (automatic-appropriate), indicating appropriate behavior in familiar settings automatic completion of activities of daily living, carryover of newly learned material, and initiation of social interactions. Individuals with a RLAS score of VII do, at times, demonstrate mildly impaired judgment and this should be taken into consideration when assessing the results of this study. The MoCA was administered on three out of the four participants. Participant 1 was unable to complete the MoCA secondary to severe visual impairment brought about by her injury. Observed scores on the MoCA ranged from 23 to 25, below the cutoff of 26 for “normal,” suggestive of mild cognitive impairment. Although all participants with TBI had previously experienced some degree of respiratory compromise (e.g., pneumonia) immediately following their injury, none reported current respiratory issues. Although it was not included officially as criterion for inclusion, all had experienced oropharyngeal dysphagia as a result of the TBI and each had received inpatient dysphagia therapy with a speech-language pathologist following injury. At the time of this study, these issues persisted for only one participant who continued to receive nutritional support via g-tube.

**Figure 1:**
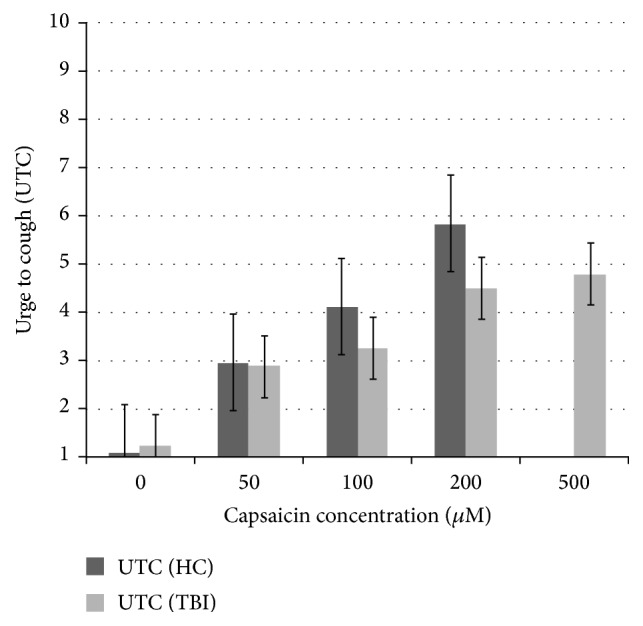
Comparison of urge to cough (UTC) ratings at increasing concentrations of capsaicin, healthy controls (HCs) compared to participants with TBI. On this scale, 1 = “no urge to cough” and 10 = “maximum urge to cough.” Participants with TBI were presented with an additional three presentations of 500 *μ*M capsaicin, for which there is no HC comparison data.

Average UTC ratings were reduced for participants with TBI relative to ratings obtained from HC participants (see Table 2 and Figure 1) at 50, 100, and 200 *μ*M capsaicin. No comparison was made at 500 *μ*M as this was not administered to HC participants.

**Figure 2:**
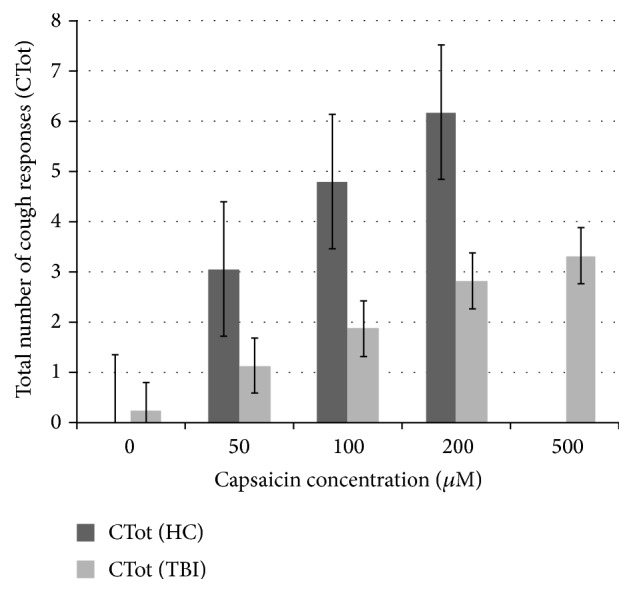
Number of cough events (CTot) recorded following presentation of capsaicin solution at various consistencies; healthy controls (HCs) compared to participants with TBI. Participants with TBI were presented with an additional three presentations of 500 *μ*M capsaicin, for which there is no HC comparison data.

Average CTot was reduced for participants with TBI relative to HC participants at 50, 100, and 200 *μ*M capsaicin (see Table 2 and Figure 2). Again, no comparison was possible at 500 *μ*M.

UTC and CTot displayed borderline-large to large effect sizes compared to the HC data (Table 3).

For the majority of HC participants (71.88%), C2 was elicited at 50 *μ*M capsaicin. In contrast, three out of the four participants with TBI required a concentration of 200 *μ*M capsaicin to elicit C2 (Table 4).

## 4. Discussion

There are a number of shortcomings in the current investigation. The results of this exploratory pilot study were obtained from a very small convenience sample of individuals with a history of TBI with tracheostomy and therefore cannot be extrapolated to include all individuals with TBI.

In spite of this, these results suggest the need for further study of dystussia resulting from TBI. Future, well-powered investigations will be better positioned to differentiate the nature and severity of these potential deficits, as well as their relative contributions to airway defense, morbidity, mortality, and quality of life. There exists growing recognition of changes to airway defense mechanisms following TBI, and a growing body of literature has elucidated the contribution of dysphagia to increases in morbidity and mortality [8–11]. However, there exists no similar body of literature addressing dystussia following TBI. Aspiration-related lung infections occur secondary to a number of factors related to both mental and physical status and deficits in swallow and cough are certainly not the sole etiology. However, because swallow and cough are mechanisms of airway protection, it stands to reason that dysfunction of either has the potential to exert potentially serious effects on airway health.

The deleterious effects of TBI on upper aerodigestive functions may be further exacerbated by posttraumatic changes to communication. It is possible that impaired attention or reduced initiation of communicative acts may prevent individuals with TBI from communicating abnormal airway sensory inputs when they occur. TBI is associated with a range of cognitive deficits to a range of modalities including attention, memory, and processing speed [5]. While stimulus-induced cough is a defensive response originating in the brainstem, cough is influenced by a range of central neural control processes, modulated by perception and attention [22, 23]. Consequently, UTC may be reduced or misinterpreted following damage of these mechanisms.

Surgical tracheostomy is commonly performed following TBI to reestablish airway access and ventilatory support. Decannulation when the tracheostomy is no longer indicated may potentially affect stimulus-induced cough [33]. Each of the participants with TBI was tracheostomized following his or her injury (although all were decannulated at least one year prior to the time of examination). Emergency tracheal intubation may also impact stimulus-induced cough reflex response [34], and all our cases were emergently intubated immediately following their injuries.

Additional factors surrounding locus of damage, lower motor neuron injury, and muscular atrophy (among others) warrant further consideration with this population. As the objective of this preliminary report was to explore the absence or presence of sensorimotor disruptions to cough in a cohort of individuals with TBI, detailed information regarding each participant's individual neurologic profile is not included.

## 5. Conclusion

The results of this study delineate clear differences in UTC, CTot, and C2 in four participants with TBI compared to HC participants. This preliminary evidence should be examined within the context of a larger, systematic study of individuals with TBI in order to refine recommendations regarding cough response screening in this population. Future investigations should attempt to discriminate if the trends observed in this investigation are applicable to a larger study cohort of individuals with TBI. If cough response deficits* are* identified in this population, additional inquiry may determine if they occur as the result of central nervous system (CNS) damage, disruption to peripheral (e.g., sensory receptive during or after cannulation) inputs, or some other combination thereof.

In spite of its role in airway protection, cough is not routinely assessed by speech-language pathologists (or other clinical professionals) who care for those with TBI and no universally accepted means exist for assessing cough function in this (or any other) neurogenic population. At present, the authors are aware of only one standardized dysphagia assessment protocol (the Mann Assessment of Swallowing Ability or MASA) [35] that includes a formal assessment of cough behavior, specifically voluntary cough. Here, patients are instructed to cough and the administering speech-language pathologist (or another specialist) judges the cough to be absent, abnormal, or normal. When cough assessment is carried out, either informally or as a component of the MASA, it relies on subjective perceptual evaluation of a voluntary motor act (“Give me a strong cough”). Although the clinician is certainly able to glean some information pertaining to strength or timing (e.g., “How strong is the patient's cough?”, “Does the patient's cough sound appropriately sharp or is it soft and breathy?”, and “Did the cough occur in close proximity to the administration of food or liquid by mouth?”) from voluntary cough assessments, limiting the focus to voluntary cough belies existing evidence of cough as a physiological function with sensory, motor, emotional/affective, and cognitive subcomponents.

Interest in UTC as an emotional state resulting from nociceptive inputs (similar to itching or the urge to urinate) is a relatively recent addition to the research literature. Cough is an extremely complex behavior and breakdown in any one portion of the cough response system could theoretically leave the airway vulnerable to aspiration and its consequences. When a speech-language pathologist witnesses uncompensated aspiration on a swallow screening or assessment, he or she is assessing the presence (or absence) of a cough response, perhaps going so far as to characterize the cough response in terms of timing or strength. While being certainly helpful with regard to issuing dietary recommendations and developing a plan of care, there is very little evidence to be gleaned as to the* mechanism* of cough impairment from standard assessments. Is there a disruption in the detection and afferent transmission of sensory inputs from the oropharyngeal or tracheal mucosa? Does the breakdown occur at the level of the cortex where cognitive awareness of the stimulation and affective response to this stimulation occurs? Do observed deficits occur as a consequence of damage to efferent (motor) pathways or muscular weakness? Unfortunately, these questions cannot be answered from our existing fund of knowledge.

Unfortunately, not all patients will have access to a comprehensive swallowing assessment by a speech pathologist. In these instances, patients who “pass” a swallow screen and are therefore not referred for further assessment may, upon closer inspection, demonstrate impairment at one point (or many points) throughout this complex sensorimotor system. The addition of cough assessment to a standardized dysphagia screen implicitly acknowledges the importance of cough as a mechanism of airway protection; elicitation of voluntary cough (as is required by the MASA) is not analogous to elicitation of cough in response to stimulation (i.e.,* reflexive cough*) due to the contribution of neurological regions (e.g., frontal lobe premotor and supplementary motor cortex) governing executive function. Additionally, from an aerodynamic perspective, stimulus-induced and voluntary cough are not interchangeable; significant differences in the rate and volume of expelled air were found in a recent investigation [36] where patients with Parkinson's disease produced both types of cough. Use of voluntary cough in isolation may in fact overestimate these measures, increasing the risk of producing a false negative screening result. It is possible that patients with dampened or absent sensory responses to cough stimulation may be able to produce a voluntary cough when instructed. Conversely, patients with deficits in motor task initiation or execution may demonstrate an intact cough response to sensory stimulation, but be unable to execute a voluntary cough maneuver on command. This disparity underscores the need for comprehensive and multimodal assessment of cough behaviors in at-risk patients as a component of routine dysphagia examination procedures.

Decreased UTC suggests impaired interoception. We present preliminary evidence of decreased UTC in a small cohort of young adults after TBI which, along with reduced CTot, could place these individuals at increased risk of aspiration and lung infection. The results presented herein are the first of their kind and the extent to which impaired sensory perception of cough stimuli is a consequence of TBI is unknown and deserves further study. Future work should (1) attempt to clarify if the observed phenomena are borne out in a larger sample of individuals with TBI and (2) determine the relative contributions of central versus peripheral nervous system structures to cough sensory perceptual changes following TBI (should they exist). Progress toward these aims will inform routine and standardized assessment of cough response behaviors which may, in turn, inform rehabilitative protocols for reducing dystussia and dysphagia symptoms and improve mechanisms of airway protection and clearance for those with TBI, potentially leading to the introduction of cough-specific screening measures. Early detection and rehabilitation of cough-related deficits in this stand to decrease injury-related morbidity and mortality while increasing quality of life following TBI.

## Figures and Tables

**Table 1:** Demographics of healthy controls (HCs) and participants with TBI.

		M/F	Age (yrs.)	Time since TBI (yrs.)	RLAS^1^	MOCA^2^	Education (yrs.)	Other PMH
TBI	1	F	49	1.26	VI	N/A	18	DVT, Asp PNA, GJ tube, double vision, and deafness
	2	M	22	2.10	VIII	23	12	Rare dysphagia episodes (thin liquids) after TBI
	3	F	20	0.55	VIII	24	12	Heterotopic ossification s/p TBI, oropharyngeal dysphagia after TBI (resolved)
	4	M	22	3.46	VII	25	15	Dysphagia s/p TBI, reporting no symptoms currently

HCs		F (*n* = 13)	23.60 (3.80)	N/A	N/A	N/A		
		M (*n* = 19)	24.19 (3.96)	N/A	N/A	N/A		

^1^Rancho Los Amigos Scale score [30]; ^2^Montreal Cognitive Assessment [31].

**Table 2:** Average (SD) subjective UTC ratings (where 1 = no urge to cough and 10 = maximum urge to cough) and total cough count (CTot) at various concentrations of capsaicin in solution obtained from healthy controls (HCs) and participants with TBI.

	Capsaicin concentration (*μ*M)				
	0	50	100	200	500
UTC (HC)	1.09 (0.31)	2.97 (1.11)	4.12 (1.50)	5.85 (1.85)	N/A^*∗*^
UTC (TBI)	1.25 (0.50)	2.88 (0.98)	3.25 (1.22)	4.50 (1.68)	4.79 (0.85)
CTot (HC)	0.00 (0.00)	3.06 (2.31)	4.81 (3.12)	6.19 (3.58)	N/A^*∗*^
CTot (TBI)	0.25 (0.5)	1.13 (0.37)	1.88 (1.07)	2.83 (0.43)	3.33 (0.72)

^*∗*^500 *μ*M capsaicin solution not administered to HC participants.

**Table 3:** Effect size calculations (Cohen's *d*) for UTC and CTot.

Capsaicin concentration (*μ*M)	Effect size (Cohen's *d*)	
	UTC	Cough production
0	−0.26	1.01
50	−0.11	−0.87
100	−0.44	−0.98
200	−0.69	−0.98

**Table 4:** Percentage of HC and TBI participants producing a two-cough threshold response (C2) at various concentrations of capsaicin in solution.

Capsaicin concentration (*μ*M)	Percentage	
	HC	TBI
0	3.13	0.00
50	71.88	0.00
100	12.50	25.00
200	9.38	75.00
